# Disinfection of Virtual Reality Devices in Health Care Settings: In Vitro Assessment and Survey Study

**DOI:** 10.2196/42332

**Published:** 2022-12-01

**Authors:** Scott C Roberts, Nancy L Havill, Rosa M Flores, Curtis Anthony Hendrix II, Maximillian J Williams, Richard S Feinn, Steven J Choi, Richard A Martinello, Asher M Marks, Thomas S Murray

**Affiliations:** 1 Section of Infectious Diseases Department of Internal Medicine Yale School of Medicine New Haven, CT United States; 2 Department of Infection Prevention Yale New Haven Hospital New Haven, CT United States; 3 Department of Pediatrics Yale School of Medicine New Haven, CT United States; 4 Department of Medical Sciences Frank H Netter MD School of Medicine Quinnipiac University North Haven, CT United States

**Keywords:** disinfection, healthcare-acquired infection, healthcare worker, virtual reality, disinfect, occupational health, occupational safety, infection control, infection spread

## Abstract

**Background:**

Virtual reality (VR) devices are increasingly used in health care settings. The use among patients has the potential to unintentionally transmit pathogens between patients and hospital staff. No standard operating procedure for disinfection exists to ensure safe use between patients.

**Objective:**

This study aims to determine the efficacy of disinfectants on VR devices in order to ensure safe use in health care settings.

**Methods:**

Three types of bacteria were inoculated onto porous and nonporous surfaces of 2 VR devices: the Meta Oculus Quest and Meta Oculus Quest 2. Disinfection was performed using either isopropyl alcohol or alcohol-free quaternary ammonium wipes. A quantitative culture was used to assess the adequacy of disinfection. A survey was separately sent out to VR device technicians at other pediatric health care institutes to compare the methods of disinfection and how they were established.

**Results:**

Both products achieved adequate disinfection of the treated surfaces; however, a greater log-kill was achieved on nonporous surfaces than on the porous surfaces. Alcohol performed better than quaternary ammonium on porous surfaces. The survey respondents reported a wide variability in disinfection processes with only 1 person reporting an established standard operating procedure.

**Conclusions:**

Disinfection can be achieved through the use of either isopropyl alcohol or quaternary ammonium products. Porous surfaces showed lesser log-kill rates than the nonporous surfaces, indicating that the use of an added barrier may be of benefit and should be a point of future research. Given the variability in the disinfection process across health care systems, a standard operating procedure is proposed.

## Introduction

Virtual reality (VR) devices are increasingly used in health care settings to benefit patients, and the examples include patients with posttraumatic stress disorder, anxiety, complex regional pain syndrome, and distraction therapy [1-4]. Recent data show the benefit expands to the pediatric population as well by reducing pain and anxiety during medical procedures through distraction [5]. VR can also be used to educate health care workers through training and simulation [6]. However, a lack of standardized cleaning and disinfection processes for VR devices has limited VR’s use in health care settings, especially during the COVID-19 pandemic [6].

Nosocomial transmission and outbreaks have been reported with many different types of medical devices in clinical use [7], and establishing standard operating procedures (SOPs) for disinfection of VR devices between patient use is paramount. One of the most common and widely sold VR headset devices worldwide, the Meta Oculus Quest 2, specifically, recommends against the use of alcohol to clean and disinfect the device in favor of antibacterial wipes due to theoretical concerns about affecting the porous material [8]. This poses challenges in clinical settings as isopropyl alcohol (IPA) is one of the most common disinfectants used on medical devices.

A protocol to clean and disinfect VR devices used in health care settings has been proposed [6]; however, no studies have quantified the efficacy of hospital-grade disinfectants on different parts of the VR equipment. Additionally, little is known about how these machines are currently disinfected in health care settings. In this mixed methods evaluation, we sought to determine the current disinfection practices in health care settings and how they were established. We also studied the effect of commonly used disinfectant wipes on the disinfection of VR headsets experimentally contaminated with common bacterial pathogens to provide evidence for the creation of an SOP to reduce infections with multipatient VR utilization.

## Methods

### Survey

To learn how health care facilities disinfect VR equipment and whether infection prevention teams are involved, a voluntary Qualtrics survey was sent via an electronic link in a group chat of 50 VR technicians working at children’s hospitals across the United States as a convenience sample.

### Ethical Considerations

The survey was approved as exempt by the Yale University institutional review board (study #2000033075).

### Laboratory Disinfection

**Figure 1:**
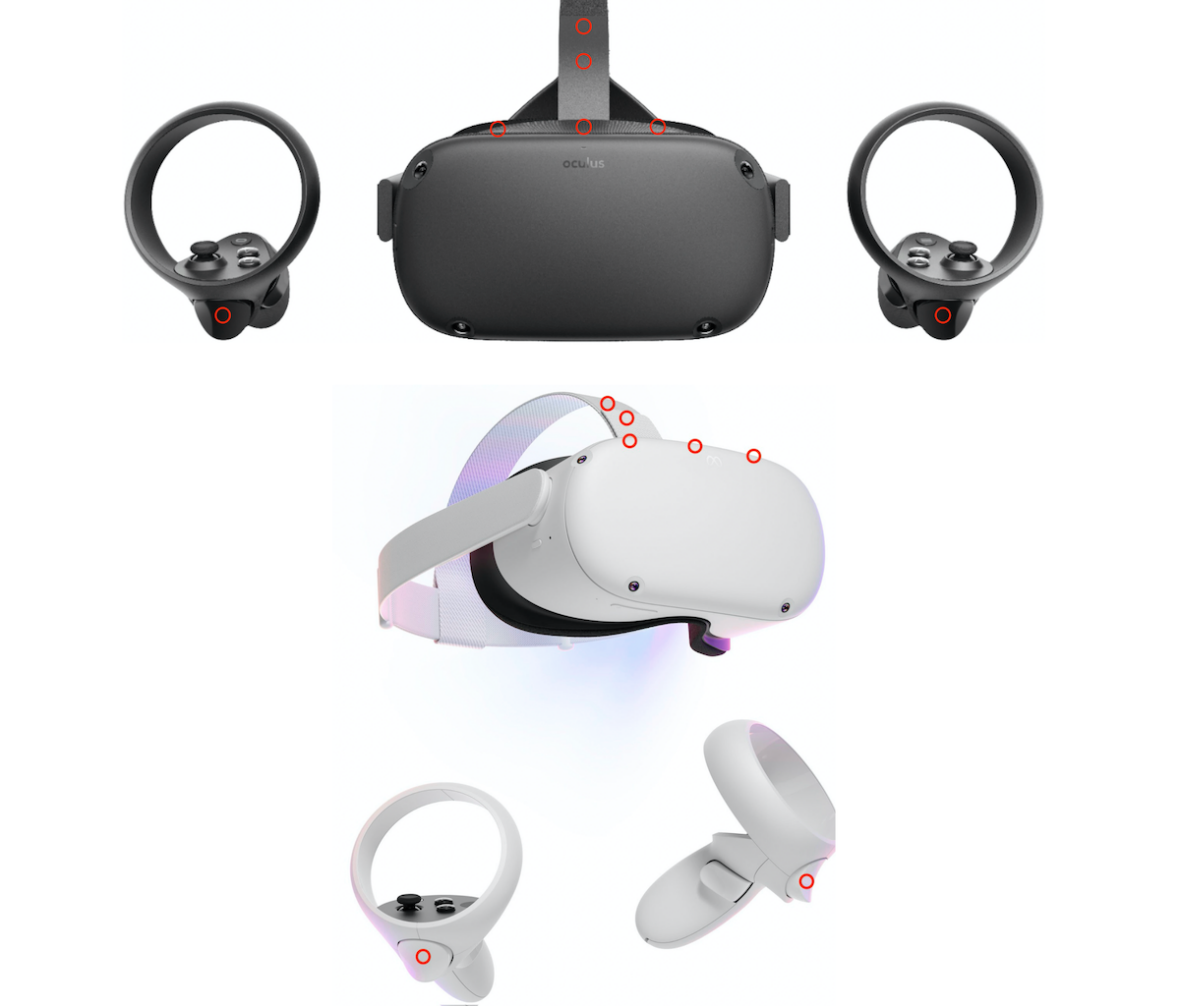
Areas where cultures were obtained (shown by red circles) of Oculus Quest (top) and Oculus Quest 2 (bottom) devices.

Three types of bacteria, *Staphylococcus epidermidis* (ATCC 12228), *Pseudomonas aeruginosa* (laboratory strain PAO1), and *Staphylococcus aureus* (ATCC 25923), were chosen because of their propensity to be present on the skin and cause infection in children with compromised immune systems. The bacteria were grown overnight in 3 mL of lysogeny broth and serially diluted to quantitate the bacterial inoculum. VR headsets and controllers were inoculated by spreading 10 μL (initial inoculum 4.1×10^6^-4.5×10^8^) onto various sites (Figure 1) and allowed to dry for 30 minutes. This large inoculum was chosen to test whether Environmental Protection Agency (EPA)–approved disinfectants achieved sufficient log-reduction in bacteria as per their instructions for use. Two VR devices were experimentally contaminated: the Oculus Quest headset and the Oculus Quest 2 headset and hand controllers (Reality Labs, Meta Platforms). These devices were chosen as these are the most popular consumer devices, the primary ones used at our institution, and contained different surface types to trial disinfection [9]. Contaminated sites included the outer surface of the headset housing on the top side, the controller buttons, and the headband straps for each device (Figure 1). These sites were chosen as they were thought to be high–touch point areas for the hands and head during patient use. We did not study the facial interface as it is our standard practice to use a disposable barrier between the facial interface and the patient’s skin. Both nonporous (Oculus Quest 1 strap, Oculus Quest 2 headset, and controller) and porous (Oculus Quest 1 headset and Oculus Quest 2 strap) surfaces were contaminated to assess disinfection efficacy.

Two products were tested for active disinfection: a 70% IPA wipe (Medium Alcohol Prep Pad, Medline) and an alcohol-free quaternary ammonium wipe (Sani-Cloth AF3 Germicidal Disposable Wipe, PDI). A positive control of inoculation without disinfection was performed for every experiment and cultured after the dry time to account for bacterial cell death from desiccation. Disinfection was performed in accordance with each product’s manufacturer’s instructions for use. For the IPA wipe, a 15-second scrub in a back-and-forth motion followed by a 15-second dry time was performed. For the alcohol-free quaternary ammonium wipe, the experimentally contaminated area was wiped to a point of saturation for 3 minutes and allowed to dry. Following disinfection, the cultures were obtained with sterile cotton swabs dipped in Dey-Engley (D/E) neutralizing broth (Hardy Diagnostics) and wiped across the entirety of the contaminated surface for 5 seconds in a back-and-forth motion. The swab was used to inoculate a D/E agar plate that was then incubated overnight at 37 C. Bacterial colony forming units (CFUs) were counted on the plates the following day. Between experiments, the entirety of the VR devices was disinfected with a 70% IPA spray to the point of saturation of the surface materials and dried overnight. The laboratory experiments satisfied the Yale University 100 CH.9 Clinical Quality Improvement criteria and were exempt from institutional review board approval.

### Statistical Analysis

The experimental design was a 3-factor crossed design with bacterial CFUs as the outcome variable. The generalized linear model with binomial distribution and logit link was used to compare whether the proportion of trials with observable bacteria counts after disinfection differed by (1) type of disinfection, (2) type of organism, and (3) surface type. All models included the natural log of bacterial count prior to disinfection and used robust standard errors. Achieving disinfection was defined as a greater than a 6-log reduction in bacterial counts. Pairwise comparisons were performed for the type of organism. Analyses were performed in SPSS (version 27; IBM Corp), and statistical significance was set at an α level of .05.

## Results

### Current VR Disinfection Practices in Pediatric Hospitals

A total of 50 VR technicians across the Unites States were invited to participate in the Qualtrics survey with a response rate of 18% (9/50). One person consented and then did not answer any of the questions. The selected results of the survey are shown in Table 1 and highlight the variability of VR use and disinfection practices.

**Table 1:** Survey of current VR^a^ disinfection practices in health care settings.

Questions and responses			Respondents (N=9), n (%)
**Frequency of VR use in the hospital**			
	<1 times per week		2 (22)
	1-3 times per week		5 (55)
	4-6 times per week		1 (11)
	Multiple times per day		1 (11)
**VR systems used in health care^b^**			
	Google Daydream		4 (44)
	Kind VR		1 (11)
	Oculus Quest 2		4 (44)
	Oculus Rift		2 (22)
	PlayStation VR		5 (56)
	Starlight Children’s VR system		6 (67)
**When disinfection is performed**			
	Before patient use		9 (100)
**Method of disinfection^b^**			
	Isopropyl alcohol		3 (33)
	Quaternary ammonium (PDI gray top)		2 (22)
	Isopropyl alcohol/quaternary ammonium (PDI purple top)		4 (44)
	Hydrogen peroxide		2 (22)
**Physical barriers used to prevent infection^b^**			
	**Any physical barriers**		
		Silicon covers	6 (66)
		Disposable eye masks	3 (33)
		Wipeable replacement head straps	2 (22)
		Hair covers	2 (22)
	No barriers present		3 (33)
**Use of standard operating procedure**			
	Yes		1 (11)
**Inclusion of the Department of Infection Prevention in establishing disinfection technique**			
	Yes		2 (22)

^a^VR: virtual reality.

^b^Some respondents gave multiple responses.

The number of VR sessions varied from less than once a week to multiple times per day. Most locations used multiple types of VR platforms with the Starlight Children’s VR system most commonly used. The Starlight Children’s VR system is a variation of the Lenovo Mirage Solo VR headset and is made of very similar materials as the Oculus Quest 1 and Oculus Quest 2. All participants noted that disinfection was performed before patient use (n=9, 100%). The methods of disinfection were variable with IPA and quaternary ammonium low-level disinfection wipes used most commonly. Most VR technicians used physical barriers between the VR device and the patient such as combinations of silicon covers, disposable eye masks, wipeable replacement head straps, and hair covers, while 3 (33%) participants did not use barriers (Table 1). Only 1 (11%) institution had an SOP for use and disinfection. Infection prevention teams were involved in assisting with VR disinfection protocols at 2 (22%) sites.

### Effectiveness of Hospital-Grade Disinfecting Wipes on VR Decontamination

**Figure 2:**
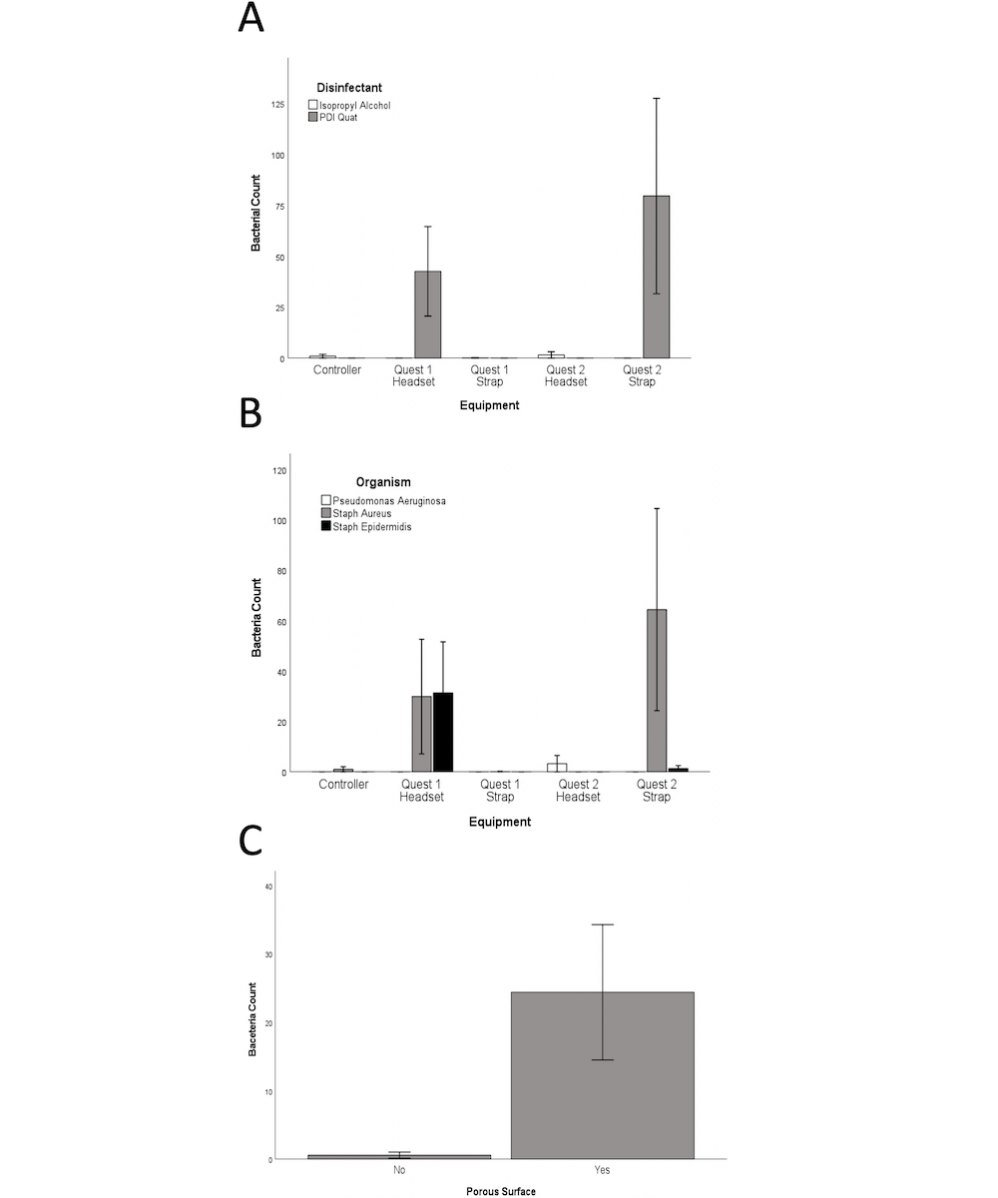
The mean (SEM) raw bacterial counts recovered after disinfection according to disinfectant and equipment (A), by organism and equipment (B), and by equipment surface type (C) are displayed. Note: Non-porous surfaces were the Quest 1 Strap, Quest 2 Headset and Controller, while porous surfaces were the Quest 1 Headset and Quest 2 Strap.

A total of 175 experiments were performed to assess disinfection (Table 2). Adequate disinfection was achieved with both the IPA and the alcohol-free quaternary ammonium wipes across all bacterial types and headset material comparing untreated with disinfected surfaces. No bacteria were recovered in 88% (154/175) of experiments. IPA wipes performed better than the quaternary ammonium wipes at reducing overall bacterial counts (*P*=.001). This difference was most pronounced on porous surfaces, where the mean quantity of bacteria remaining after the alcohol-free quaternary ammonium use was more than after IPA use (Figure 2A).

When comparing the disinfection by an organism, there were more CFUs of *S aureus* and *S epidermidis* recovered after attempted disinfection than *P aeruginosa* (*P*=.05 and *P*=.03, respectively), with this difference most pronounced on porous surfaces (Figure 2B). There was no significant difference between the recovery of *S aureus* and *S epidermidis* (*P*=.72). Finally, fewer bacteria were recovered from porous surfaces after inoculation but prior to performing disinfection. However, more bacteria were recovered after disinfection from porous surfaces than from nonporous surfaces (*P*=.01) as shown in Figure 2C, confirming that porous materials inoculated (Figure 1) were more difficult to disinfect because of their availability to absorb the bacteria-containing liquid.

**Table 2:** Bacterial count by disinfection method, organism, and surface.

Factors		Observations, n	Bacterial count					
			Mean (SD)	Median	Minimum, Maximum	% Zero^a^		
**Disinfectant**								
	Isopropyl alcohol	94	0.60 (4.52)	0	0, 42	97		
	Alcohol-free quaternary ammonium	81	23.12 (82.4)	0	0, 500	88		
**Organism**								
	*Pseudomonas aeruginosa*	51	0.88 (6.06)	0	0, 42	98		
	*Staphylococcus aureus*	80	17.46 (76.5)	0	0, 500	86		
	*Staphylococcus epidermidis*	44	9.43 (41.2)	0	0, 265	82		
**Surface^b^**								
	Not porous	100	0.57 (4.4)	0	0, 42	97		
	Porous	75	24.4 (84.5)	0	0, 500	77		

^a^The percentage of times when 0 bacterial colony-forming units were observed.

^b^Nonporous surfaces were the Quest 1 Strap, Quest 2 Headset, and controller, while the porous surfaces were the Quest 1 Headset and Quest 2 Strap.

## Discussion

Adequate disinfection of VR devices can be achieved through the use of low-level EPA-approved hospital-grade disinfectants commonly used in clinical settings for devices that are exposed to intact skin, including IPA and quaternary ammonium wipes. We found a greater than 6-fold logarithmic reduction from initial bacteria inoculation across all pathogen types and VR device surfaces when using either product. However, we did observe the differences when evaluating raw-bacterial counts after disinfection. Notably, IPA performed better than the quaternary ammonium wipe, particularly for porous surfaces. It is possible that IPA penetrates porous surfaces better than quaternary ammonium products due to the vigorous 15-second scrub, and future studies should evaluate how well different wipes perform on these types of surfaces. We also observed that *S aureus* and *S epidermidis* persisted on surfaces at greater densities than *P aeruginosa*, possibly reflective of a mechanism in strain type or environmental survivability. Finally, and perhaps most critically, we observed lower bacterial counts after inoculation but before disinfection, and greater bacterial counts after disinfection, on porous surfaces when compared to nonporous surfaces. This suggests that bacteria may be entering the pores in the material, potentially reducing exposure to the disinfection material. Additionally, using swabs to recover bacteria from porous surfaces is suboptimal as we do not recover bacteria that have penetrated deeper into the material as well as nonporous surfaces which have better transfer efficiency [10]. Thus, despite consistent recovery after disinfection from porous surfaces in these experiments, we likely have overestimated the efficacy of disinfection for this material. Of note, there was outstanding disinfection of all nonporous surfaces, making it the preferred material for the construction of VR devices in health care. Manufacturers should consider material in the design of both headsets and straps, and our data support the use of nonporous material, particularly in health care settings where persistent bacteria may serve as a nidus for transmission to the next VR device user. If porous surfaces are present, there should be adequate barrier protection to prevent the transmission of microbes. This also then allows for the use of IPA without concern for damage to any porous components of the device.

Nosocomial transmission and outbreaks associated with the use of medical devices are well documented and a primary concern for using VR devices in health care settings [7]. Consequently, facilities may restrict VR devices from patient use out of concern. We found substantial variability between facilities in the frequency of device use, disinfection method, and barrier protection used. Importantly, almost all sites reported that infection prevention teams were not involved in performing a risk assessment for device use during patient care, and SOPs for disinfection were absent in all but one institution. Establishing a standard process that appropriately disinfects VR devices to allow for safe and expanded use in health care settings while avoiding equipment degradation can benefit patients and health care workers alike. It is critical to ensure that when new devices such as VR equipment are introduced into patient care settings, Infection Prevention and other stakeholders are involved prior to the purchase of the devices to ensure there is an acceptable plan for device reprocessing.

Based on this generated data set, manufacturer’s instructions for use [8], health care infection prevention best practices, and previous literature or expert opinion [6], we propose an SOP for VR use and disinfection in the health care setting (Textbox 1). This is particularly important as the patient population served may be undergoing chemotherapy or other immunosuppressants that can increase the risk of infection.

**Suggested standard operating procedure for the disinfection of virtual reality devices.**

**Before use**

- Avoid on patients with nonintact skin or active infections on the head or hands that cannot be covered and might come into contact with the device
- Avoid use on patients known to be colonized with pathogens where specialized disinfection is required, including *Clostridioides difficile*, *Candida auris*, *Mycobacterium tuberculosis*, and nonenveloped viruses
- Patient and staff perform hand hygiene
- A nonporous cover over the face pad, a disposable face pad cover, or both should serve as a barrier between the patient’s face and the device. Hair should also be covered (eg, bouffant and washable cloth surgical cap). Any porous material that makes contact with the patient’s skin or hair should be covered with a barrier
- Devices should be assessed for alcohol compatibility. If the device is not alcohol compatible, a nonalcohol-based disinfectant should be used
- Perform disinfection with a device compatible Environmental Protection Agency–registered product List H [11] according to the manufacturer’s instructions for use, ensuring that all surfaces of the headset (including the strap, the casing, the inner and outer facepieces, and the lens), the controller, and the nonporous, nondisposable face cover are saturated. Do not use wipes on multiple devices.

**After use**

- Patient and staff perform hand hygiene
- Staff don appropriate personal protective equipment, which should include nitrile gloves at a minimum unless other personal protective equipment is required per the patient’s transmission-based isolation precautions
- Remove the device from the patient and placed on a clean disposable pad
- Discard the disposable face cover, if present
- Remove nonporous, nondisposable face cover from the device, if present
- Clean all visibly soiled areas with disposable wipes or paper towels
- Repeat disinfection as above
- Allow headset and controllers to dry according to the product instructions for use
- Store the device in a dry space physically separated from nondisinfected devices
- Patient and staff perform hand hygiene

In creating the SOP (Textbox 1), we considered VR devices a noncritical item requiring low-level disinfection between patients because they are most commonly exposed to intact skin only. Given disinfection success with the IPA and alcohol-free quaternary ammonium wipes, we suspect other equivalent low-level disinfectant products (eg, combination IPA or quaternary ammonium wipes) would be adequate, especially when applied to nonporous services [12]. We did not evaluate high-level disinfectants or sterilization procedures that may be required in the event of device exposure to nonintact skin or mucous membranes. We suggest avoiding VR device use on patients who have breaks in the skin on the hands or head region that cannot be appropriately covered and could come into contact with the device, thus avoiding the need for a high level of disinfection.

As disinfection was successfully achieved for a variety of pathogens, VR use is most likely safe for patients where contact isolation (gowns and gloves) is required in the hospital, including patients colonized with methicillin-resistant *S aureus* or vancomycin-resistant Enterococci. However, we suggest clinicians exercise caution when using VR devices with patients colonized with harder-to-eradicate pathogens such as *Clostridioides difficile*, *Candida auris*, and nonenveloped viruses where sodium hypochlorite or other high-level disinfection methods may be required. The pathogens tested (*S aureus*, *P aeruginosa,* and *S epidermidis*) are very common organisms in health care settings seen in both the adult and pediatric populations, and these results from disinfection are likely to be applicable to most health care settings, regardless of the patient’s age.

Limitations of this study include the single-site nature limiting generalizability and the poor survey response rate. We also only tested 2 VR devices from the same company and 2 methods of surface disinfection. Preliminary experiments with an ultraviolet C (UVC) device specifically designed to decontaminate VR devices failed to produce adequate disinfection (data not shown). Since UVC disinfection depends on the angle and distance from the surface to the UVC source [13], the geometry of the headsets may make UVC disinfection more challenging. Further studies evaluating alternative disinfection methods, including UVC and other types of VR devices, are ongoing.

## Abbreviations

CFU: colony forming unit
D/E: Dey-Engley
EPA: Environmental Protection Agency
IPA: isopropyl alcohol
SOP: standard operating procedure
UVC: ultraviolet C
VR: virtual reality

## Data Availability

The data sets generated and analyzed during the current study are available from the corresponding author upon reasonable request.
